# Toward a unified gait freeze index: a standardized benchmark for clinical and regulatory evaluations

**DOI:** 10.3389/fneur.2025.1528963

**Published:** 2025-05-08

**Authors:** Alessandro Schaer, Henrik Maurenbrecher, Carlo Mangiante, Roman Sobkuliak, Kathrin Müsch, Paula Sanchez Lopez, Eduardo Martin Moraud, Olgac Ergeneman, George Chatzipirpiridis

**Affiliations:** ^1^Magnes AG, Zurich, Switzerland; ^2^NeuroRestore, Defitech Centre for Interventional Neurotherapies, Centre Hospitalier Universitaire Vaudois (CHUV), University of Lausanne (UNIL), and Ecole Polytechnique Fédérale de Lausanne (EPFL), Lausanne, Switzerland; ^3^Department of Clinical Neurosciences, Lausanne University Hospital (CHUV) and University of Lausanne (UNIL), Lausanne, Switzerland

**Keywords:** Parkinson's disease, freezing of gait, freeze index, gait analysis, medical device regulation, telemonitoring

## Abstract

Freezing of Gait (FOG) is a disabling motor symptom that affects a majority of individuals with advanced Parkinson's disease, severely limiting mobility, independence, and quality of life. Automatic methods for detecting FOG using the freeze index (FI) have been widely proposed to systematically monitor FOG in real life and guide therapy optimizations. However, methods to estimate the FI have relied on a broad range of measurement technologies and computational methodologies, often lacking mathematical rigor. The inconsistency across studies has made it difficult to directly compare results or draw definitive conclusions. This lack of standardization has severely hindered the acceptance of FI by regulatory agencies as a reproducible, robust, effective and safe measure on which to base further developments. In this study, we formalize the definition of the FI and propose a rigorous, explicit estimation algorithm, which may serve as a standard for future applications. This standardization provides a consistent and reliable benchmark. We also provide an overview of existing FI estimation methods, discuss their limitations, and compare each one of them with the proposed standard. Our method demonstrates improved performance compared to existing approaches while effectively mitigating the risk of divergent outcomes, which could otherwise lead to unforeseen and potentially hazardous consequences in real-world applications. Our algorithm is made available as open-source Python code, promoting accessibility and reproducibility.

## 1 Introduction

Parkinson's disease (PD) is a neurodegenerative disorder affecting an estimated 8.5 million people worldwide.^1^ Over the past 25 years, the prevalence of PD has doubled, with an estimated 329,000 deaths attributed to the disease in 2019 alone. PD is primarily characterized by the progressive degeneration of dopaminergic neurons in *subtantia nigra*, resulting in reduced dopamine levels. This dopamine deficiency leads to hallmark motor impairments such as tremor, rigidity, bradykinesia, and postural instability along with gait disturbances including shuffling steps, stooped posture, difficulties with gait initiation (1, 2).

Over half of advanced-stage PD patients also experience freezing of gait (FOG), which are episodes lasting up to few seconds where the patient is unable to move, despite the intention to do so (3). Although FOG predominantly manifests in advanced stages, it also affects about 26% of the patients in early stages (4). The combination of frequency and severity of FOG events is closely linked with the state of the disease, making FOG a promising biomarker for early diagnosis and intervention planning (5). However, the unpredictability of FOG episodes poses challenges for clinicians in accurately assessing their nature and frequency during daily life. Automated FOG detection in real-world settings could help clinicians evaluate and refine treatment strategies, better pinpoint the underlying mechanisms, and ultimately reduce FOG episodes and improve quality of life of PD patients.

Today, one of the most used methods for detecting FOG is the freeze index (FI), first introduced in 2008 by Moore et al. (4). While several machine learning based methods for the detection of FOG have been proposed in the literature (5–11), culminating in a contest to further promote the research in this direction (12), the FI still provides an interpretable alternative, due to its definition based on first-principles. This method originates from the spectral analysis of gait signals recorded from pressure insoles (13). As FOG episodes are characterized by trembling legs, the FI tracks the relative amount of high frequency components in the sensor signals, with its value increasing whenever the dominant signal energy gets shifted toward higher frequencies. The FI is not a direct measure of FOG, as it only relates the relative amount of freeze-frequencies content in sensor signals. Therefore, it can result in false positives by non-FOG events living in the same frequency band as FOG, when a threshold is applied to it in order to obtain a FOG classifier. Albeit limited (6, 14), the FI represents an interpretable biomarker linked to FOG, which makes it interesting in a regulated, clinical setting. It should be seen as one more datum in the monitoring of a complex disease such as PD.

Since its inception in 2008, the FI has been used extensively in subsequent studies to detect FOG events using body worn inertial sensors (15–19). However, despite its well-accepted use in research protocols, its implementation has shown significant differences across studies, many of which suffer from a lack of rigorous mathematical foundation or omit key technical details. This lack of standardization, makes accurate replication challenging. Furthermore, this heterogeneity leads to complications in the use of the FI across institutions, and hinders its widespread adoption for large multi-centric clinical studies. From a regulatory standpoint, the risk of divergent outcomes resulting from these inconsistencies makes it impossible to refer to *the* FI for any certified medical device (MD). Currently, the variability between methods is unacceptable for regulatory approval. This hinders the use of the FI for FOG (remote) monitoring in PD patients further, as the FI computed FI values depend on the used definition, and consequently so do any inferences about FOG from the FI itself.

To address these shortcomings, here we establish a solid theoretical and practical framework for FI estimation that allows for consistent and reproducible measurements, finally making the FI an established, matured metric. Despite its limitations, such as voluntary stops and turns (14, 20), we are convinced by the value of the FI for monitoring FOG and PD among other measurements, such as gait assessments and cognitive tests. However, this work is focused on establishing robust FI estimation, but makes no claims about the FI's predictive validity for FOG events. We aim to promote its future use as a digital biomarker for PD progression and intervention planning. In this research, we make four primary contributions:

1. We provide an overview of existing FI estimation methods followed by quantitative comparisons that highlight the degree of divergence between these approaches.
2. We present a formal mathematical definition of the FI and provide a rigorous implementation method that is efficient, reproducible and easy to implement. This formalization lays the foundation for the use of the FI as part of approved gait monitoring systems.
3. We conduct a comprehensive comparative analysis between our proposed standard and other implementations found in literature. The proposed FI estimation method provides estimates that outperform previous definitions on Gaussian signals and are comparable on real-world data.
4. We enhance accessibility and reproducibility by providing open-source Python code implementing our FI estimation method and reviewed methods. This opens new avenues for the FI to be implemented as part of gait-analysis metrics embedded in medical devices, further extending the possibilities for telemonitoring of FOG in PD patients.

## 2 Methods

In the context of this work we investigate the definitions of the FI found in (4, 15, 16, 18). Henceforth, we shall refer to these as: Moore for the definition found in (4), Bachlin for the definition found in (15), Zach for the definition found in (18), and Cockx for the definition found in (16).

### 2.1 Existing descriptions: a brief history of the FI

In 2003, the idea of spectral analysis of gait signals—in particular insole forces—was introduced in (13). In this paper the authors draw the attention to the larger power in the 3–6 Hz frequency band during freezing compared to the 0–3 Hz band during locomotion. This concept was then extended in (4), which introduces the Moore FI in 2008, where the freeze band is extended to the range 3–8 Hz. Unfortunately, the authors only provide a qualitative description of the FI, lacking a formal mathematical formulation and thus leaving room for interpretation. This makes it prone to subjective evaluation, which is not desirable for any objective measure. The provided “verbal” description of the method leaves some questions unanswered, e.g. which spectral estimation method is to be used and what preprocessing steps are to be applied. Moore also introduces threshold values to discriminate between FOG and non-FOG events based on the FI value. Both a personalized value based on the first two statistical moments of the FI during standing and a global threshold are introduced. To be able to apply the global threshold though, the authors rely on a normalization of the FI. The procedure there described can be formally defined as: $n_{M}:{\mathbb{R}}^{+}\to \mathbb{R},x\mapsto n_{M}\left(x\right)=\ln\left(100\cdot x\right)$, where ln denotes the natural logarithm. We refer to *n*_*M*_ as the *Moore normalization*.

By analyzing Moore's definition, it becomes clear that the concept of the FI comes with a number of hyperparameters, namely the signal sampling frequency, the time horizon (window width), the choice of the proxy signal (i.e. the signal from which the FI is estimated), the window preprocessing methods, the power spectral density (PSD) estimation method, and the normalization function. In addition to these, the threshold value can be considered as a further parameter, if one wants to use the FI to classify FOG events in a binary fashion. Many of these aspects are not investigated any further in the literature.

In the following years, the FI was used in further research, spawning multiple varying implementations. In 2009, the Daphnet dataset was created and inspected using the Bachlin FI, which features a different time horizon, sampling frequency, and normalization function. In 2013, the authors of the original FI definition published another paper on the subject (17) using a different configuration compared to the one of their original Moore definition, in particular a different locomotion frequency band is used, and different time horizons. By 2015, the FI is referred to as a “validated algorithm” by Zach, and is used by the authors to evaluate FOG events from accelerometer data, but the FI is computed using the authors' own choices for time horizon and proxy signal, while leaving the preprocessing steps, the spectral estimation method, and the normalization function unspecified. In 2023, the FI was put to the test compared to heart-rate measurements (heart-rate z-score as proxy) using Cockx's definition, once more using different time horizon, sampling frequency, proxy signal, and preprocessing steps.

### 2.2 Limitations of the existing definitions

There does not seem to be an agreement in the existing literature on the FI, let alone an investigation on the effects of the hyperparameters which limits the reproducibility of the results and the adoption of the FI as a standardized digital biomarker. The FI has been described in words, appears to be used as a concept, but lacks a formal definition: it appears that the literature could not yet agree on a single definition of the FI, nor an algorithm to estimate it. The evolution through time in terms of scientific literature and the variety of hyperparameters used for the estimation of the FI is summarized in Table 1. It appears further, that standard good practices of signal analysis, such as detrending of windows and tapering (21), are not rigorously applied. In Bachlin the proxy window is detrended by its average and in Cockx Hann windowing is used for tapering, but no source was found applying these methods in the correct and complete sequence.^2^ Additionally, only acceleration signals have been considered, despite the original analysis being carried out on vertical ground reaction forces (13). There seems not to be any agreement on the location of the accelerometers and on the best signal (direction) to be used, suggesting that the choice of the proxy signal might not be critical. A comparison of various proxy choices is included in this paper.

**Table 1:** The history of the computation of the FI.

**ID**	**Moore**	**Bachlin**	**Moore13**	**Zach**	**Cockx**
Reference	(4)	(15)	(17)	(18)	(16)
Year	2008	2009	2013	2015	2023
Motion band [Hz]	0.5–3	0.5–3	0–3	0.5–3	0.5–3
Freeze band [Hz]	3–8	3–8	3–8	3–8	3.5–8^†^
Window *T* [s]	6.0	4.0	7.5, 10	2.0	3.0
Step size [s]	Not specified	0.5	0.2	0.1	1/256
Samp. freq. [Hz]	100	64	100	100	256
Proxy signal	Shank vert. acc.	Shank vert. acc.	Shank acc.	Lumbar fwd acc.	Shin vert acc.
			Thigh acc.		
			Lumbar acc.		
Preprocessing	Not specified	Mean μ(*x*) subtraction	Not specified	Not specified	Hann window *w*
PSD	Not specified	|FFT(*x* − μ(*x*))|^2^	Not specified	Not specified	|FFT(*x* · *w*)|^2^
Normalization	Moore *n*_*M*_	None	Not specified	Not specified	Moore *n*_*M*_
Personal ths.	μ(FI_standing_)+0.1·σ(FI_standing_)	1.5 | 3.0^*^	Not specified	μ(FI_walking_)+2·σ(FI_walking_)	Not specified
Global ths.	2.3	Not specified	3.0	1.47	Not specified

For the sources where various combinations have been experimented with, only the best predictor configuration options are listed. acc., acceleration; fwd, forward; samp. freq., sampling frequency; ths., threshold; vert., vertical.^*^These values were found in the original MATLAB code of (15).^†^This is the range reported in the paper (16), but in the annexed Matlab code the range 3–8 Hz is used instead, as of 18.09.2024: https://github.com/helenacockx/FI-HR_duringFOG.

The FI has not been unambiguously defined yet—in the remainder of this paper, a formal FI computation method is introduced, and its performance is compared to the plurality of definitions present in the literature, whose original meaning was replicated to the best of the authors' intentions. It is the authors' opinion that the existing literature falls short on the actual definition of the FI from a mathematical, algorithmical and a signal-processing perspective. To summarize the inconsistencies, the presented FI implementations (4, 15–18) have shown:

- No agreement on the time window size *T* (values range from 2 to 10 s);
- No agreement on the necessary preprocessing steps (average subtraction in Bachlin and Hann tapering in Cockx are seen, but neither seem complete nor fully satisfying good practices);
- No questioning of the quality of the PSD estimates used [the square magnitude of the FFT is used most often, but it is known that spectral estimation is no trivial feat (22)];
- No discussion on the frequency resolution requirements in the frequency domain: various sampling frequencies and window sizes have been employed for the time-frequency analysis.
- No agreement nor analysis of the effects of the definition of the locomotion (0–3 Hz and 0.5–3 Hz have been used) and freeze (3–6 Hz, 3–8 Hz, and 3.5–8 Hz have been used) frequency bands;
- No agreement on the sensor placement (shank, thigh, lower back);
- No agreement on the proxy signal [vertical acceleration, forward acceleration, after the original work was done on (vertical) ground reaction forces (13)];
- No investigation on the use of other (inertial) signals, such as angular velocities, which would naturally be unbiased by gravity;
- No agreement on the scaling of the FI (Moore scaling seems to dominate, but no scaling is mentioned in Bachlin, for example);
- No agreement on thresholds for discriminating FOG from non-FOG (a global threshold of 2.3 is used in Moore and 1.47 is brought forward in Zach, however these attempts do not fully resolve the issue, as differences and historical trends indicate a lack of universal threshold);
- No cross validation of the thresholds (neither the personalized nor the global values appear to have been determined using all data for “training” and maximize some performance metric, but no “testing” appears to be done on unseen data).

The reigning disagreement in literature means that the FI cannot be considered a digital biomarker for FOG until consensus on the evaluation of the FI is reached or as long as the differences in the methods have not been quantified. Our contribution in this sense is twofold. First, we argue that a formal definition of the FI, based on good signal processing practices and rigorous formulations, alongside a comparison of the proposed definition and implementation with the existing definitions is a void in literature that ought to be filled. The standardization of the FI enables its use in MDs and its use as an established digital biomarker for FOG. Second, we provide a quantitative comparison of the existing definitions of the FI. These efforts are meant to establish solid foundations for the future use of the FI as a digital biomarker and the study of its relation to FOG, not only based on thresholding.

### 2.3 Formalization

We introduce a formal definition for the FI, starting from continuous-time, and moving then to computable, robust estimations for discrete-time signals using multitaper spectral estimation. The formalism is limited to the computation of the FI itself, and does not cover its use as FOG classifier. This latter aspect is to be investigated in a clinical trial based on the formalism introduced herein.

It shall also be noticed that the introduced formalism does not aim at minimizing the FI's intrinsic limitations, such as its sensitivity to non-FOG motions sharing the same frequency band such as turns and voluntary stops. We justify this choice by valuing the FI's simplicity and interpretability—given additional (sensor) information it should be possible to discriminate confounding events upstream. For example, gyroscopic signals could be used to classify turns and distinguish them from false positive FOG event detections based on the FI. All of this is beyond the scope of this work.

#### 2.3.1 Continuous time

Let *x*(*t*) be real-valued, continuous-time signal. Let *w*(*t, T*) be a window signal of width *T* centered at 0—if not specified further, let *w*(*t, T*) be the rectangular window


$$\begin{array}{l} w\left(t,T\right)=\{\begin{array}{c} 1\text{ if }|t|\leq T/2,\\ 0\text{ otherwise.} \end{array} \end{array}$$

Let PSD(*f*; *x, w, t, T*) be the power spectral density at frequency *f* of the signal *x*(*t*) windowed with *w*(τ−*t, T*), i.e.


$$\begin{array}{l} \mathrm{PSD}\left(f;x,w,t,T\right)=|X\left(f;w,t,T\right){|}^{2}, \end{array}$$

with *X*(*f*; *w, t, T*) being the Fourier transform of *x*(*t*) windowed with *w*(τ−*t, T*)


$$\begin{array}{l} X\left(f;w,t,T\right)=\int_{-\infty }^{+\infty }x\left(\tau \right)\cdot w\left(\tau -t,T\right)\cdot e^{-j2\pi f\tau }d\tau . \end{array}$$

Further, let *f*_freeze_(*f*_*t*_) = [*f*_*t*_, 8] Hz and *f*_loco_(*f*_*t*_) = [0.5, *f*_*t*_] Hz denote the freezing frequency band and the locomotion frequency band respectively, and *f*_*t*_ denote the locomotion-freeze threshold frequency. We then formally define the freeze index at time *t* to be


(1) $$\begin{array}{l} \mathrm{FI}\left(t\right)=\ln\left(100\frac{\underset{f_{\text{freeze}}}{\int }\mathrm{PSD}\left(f;x,w,t,T\right)df}{\underset{f_{\text{loco}}}{\int }\mathrm{PSD}\left(f;x,w,t,T\right)df}\right). \end{array}$$

It shall be noted that this formal definition deviates from the original outlined in (4) as the square of the ratio is not taken. The rationale behind this choice is that, as the Moore normalization is part of the definition, the squaring of the fraction would only lead to a scaling by the factor of two in the end value. More on this can be found in Section 2.3.3.

#### 2.3.2 Discrete time

It shall be noted that in practice, the definition of the FI introduced in Equation 1 can only be estimated. This is due to the discrete nature of sampled signals, for which the Fourier transform is replaced by estimation methods such the fast Fourier transform (FFT) (23) or Welch's method for PSD estimation (24), and integrals are approximated with numerical methods, such as trapezoidal integration. This is particularly relevant for a clinical applications, as this context focuses more on the estimation (as an indicator of a clinically relevant outcome) than its formal definition. In the remainder of this paper we will focus on the methods to estimate the FI and on the comparisons of these methods.

#### 2.3.3 Multitaper FI estimation algorithm

We propose an FI estimation algorithm based on the short-time Fourier transform (STFT) and multitaper spectral estimation (25), see for example (22) for a review on the subject. An attempt is made toward providing a rigorous computation scheme and at the same time avoid unnecessary operations. Open source Python code leveraging the SciPy library (26) is available at https://github.com/magnesag/freeze-index.

**Figure 1:**
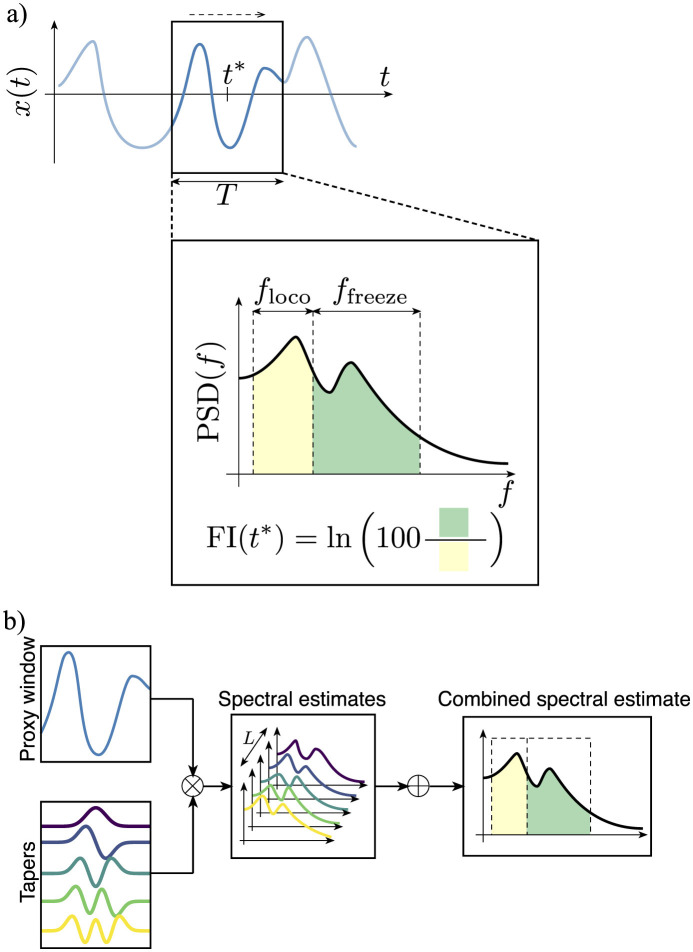
FI estimation problem and multitaper estimation algorithm conceptual visualization. **(a)** Given the gait (proxy) signal *x*(*t*), the FI is computed for each time t^*^ over the sliding time window of duration *T*. Since FOG manifests in higher frequency bands (*f*_freeze_) than regular locomotion (*f*_loco_), the FI for said window is defined as the ratio of the freeze band area (green area on the right) to the locomotion band area (yellow area on the left). **(b)** The multitaper method is used for estimating the power spectral density (PSD) of each sliding window, as it provides better estimates than other methods (22).

We formalize the estimation of the FI by the following algorithm. Let *T* be the time window in seconds, and *f*_*s*_ the sampling frequency in Hertz. Let *x*(*t*) = {*x*[*k*]} be a continuous-time, real-valued signal with *N* samples, taken at time *t* = *k*/*f*_*s*_  with  *k* = 0, 1, 2, ..., *N* − 1. Let the Fourier transform *X* of *x*(*t*) be evaluated by the FFT [X = fft(x(t)), see (23)], as part of the STFT spectrogram() function call. Let [*z*] denote the rounding operation, i.e. [*z*] = ⌊*z* + 0.5⌋. Let *W* = (*w*_1_, ⋯ , *w*_*L*_) be the first *L* Slepian windows (27) of appropriate size and let DPSS() be a method to generate such windows. Furthermore, let trapz(y, x) be the numerical integration of y along x using the trapezoidal rule. Let *f*_loco_(*f*_*t*_) = [0.5, *f*_*t*_]*Hz* and *f*_freeze_(*f*_*t*_) = [*f*_*t*_, 8]*Hz* be the locomotion and freeze intervals as a function of the threshold frequency *f*_*t*_ respectively. Let MAF(*x, M*) denote the moving average filter of width *M* being applied to signal *x*. The proposed FI estimation method is described in Algorithm 1. In Figure 1, we illustrate the estimation problem at hand as well as the idea behind multitaper spectral estimation.

**Algorithm 1:**
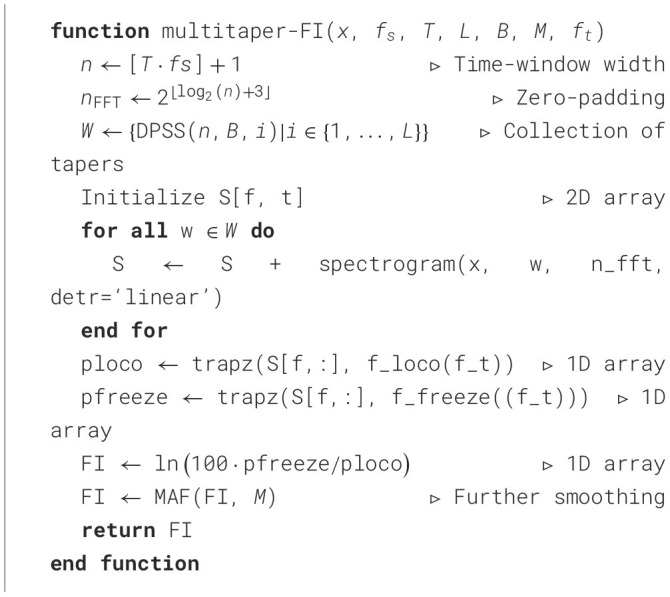
Multitaper FI estimation algorithm.

The proposed Algorithm 1 features the following properties/steps:

1. Detrending of the window using linear-dedrifting.
2. Zero-padding for increased frequency resolution and efficient FFT evaluation.
3. Multi-tapering PSD evaluation with orthogonal (Slepian) tapers (22, 27).
4. Numerical integration of the PSD via trapezoidal method.
5. Moore scaling of the FI.
6. Moving average filtering of the estimated FI signal.

It shall be noted that none of the existing FI implementations features this complete set of steps. Opposed to the existing FI implementations, the algorithm does not perform:

1. Scaling of the FFT, as it is unnecessary due to the ratio being computed.
2. Division by *L* in the averaging of the tapered spectra, as the computation of the ratio makes this operation redundant too.
3. Squaring of the ratio, as it only represents a scaling of the FI by a factor 2 in the log-scale after application of the Moore normalization step (log(*x*^2^) = 2log(*x*)). This is deemed unnecessary as:

(a) The methods found in literature all define their own thresholds for distinguishing between walking and freezing (4, 15, 18);
(b) Personalized thresholds might even be needed for better performance.

Hence, the actual (absolute) value of the FI is not as important as its relative change within the time series.

### 2.4 Comparing definitions

#### 2.4.1 Performance evaluated on theoretical results

We compare the FI definitions for signals with well-defined PSDs for which a theoretical FI value can be computed explicitly. Gaussian white noise n(t)~N(μ,σ) is such a signal. Gaussian white noise is characterized by a constant PSD (28), hence for the FI definition introduced in Equation 1 we obtain for *f*_*t*_ = 3*Hz*


(2) $$\begin{array}{l} {\mathrm{FI}}_{n,\text{theory}}=\ln\left(100\frac{8-f_{t}}{f_{t}-0.5}\right)=\ln\left(200\right)\approx 5.30. \end{array}$$

We repeat this exercise on all existing FI definitions to find the theoretical FI values for white noise. These are found to be 5.99 for Moore and Zach, 2.00 for Bachlin, and 5.78 for Cockx. In the ideal case, the FI computed on white noise should be constant at all times, as its PSD is time invariant, i.e. all time-domain slices are per definition independent and feature the same spectrum. Since white noise is a mathematical construct, we evaluate the theoretical performance by the following two metrics:

1. The variability of the FI, measured by the standard deviation of the FI signal evaluated on a simulated white noise approximation signal.
2. The root mean squared error (RMSE) of the FI signal from the theoretical value.

We generate the white noise signal using Numpy's np.random.randn() (29). To minimize the undesired effects of randomness we:

- Evaluate all methods on the same input signals;
- Repeat the evaluation with *M* = 10 randomly generated signals.

We then report the average and standard deviation of the metrics for the *M* trials. The process is repeated for sampling frequencies *f*_*s*_ 64 Hz, 100 Hz, and 256 Hz which are the sampling frequencies found in (4, 15, 16, 18). For a fair comparison with the existing definitions, the moving average filter in the proposed multitaper implementation is deactivated for this evaluation.

#### 2.4.2 Sample data comparison

While it is clear that the existing methods were not rigorously introduced and lack standardization, it is also true that any new method should at least be comparable to the existing consensus. We compare the various FI definitions on data from the Daphnet dataset (15). We focus on the FI solely, and not on its quality as a FOG classifier, as this would require a thorough discussion and analysis on the choice of threshold on a generalizable dataset while potentially also controlling for the FI's intrinsic limitations, which is outside the scope of this work. It shall be noted that the used dataset is limited to only ten participants in a semi-controlled environment, thus generalizability in terms of the use of the FI as measure for FOG cannot be achieved. Albeit limited for FOG detection, the used dataset provides a benchmark for the estimation methods in terms of FI, while keeping all sources in the public domain, easing reproducibility and cross-verification. Arguably, generalizability of the FI itself should be dataset agnostic, as it is a purely signal processing process, without direct clinical output. We compare the FI estimation methods on the real-world data using the similarity analysis described in details in Section 2.4.3.

While not all FI definitions have been used on data sampled at 64 Hz, it is argued that sound estimation methods should be robust against sampling frequency choice. Conversely, one can argue that a method's sensitivity to sampling frequency is an indicator of poorer quality. Hence, we proceed with our analysis using the public dataset without further adjustments.

#### 2.4.3 Similarity analysis

Mostly due to differences in step size of the windows (i.e., number of samples by which the window is slid), FIs computed on the same input will generally not feature the same number of samples when two different definitions are used for the evaluation. Hence, the FI signals of the different methods cannot be compared on a point by point basis. Therefore, the FI signals are resampled to the same number of samples using linear interpolation to compute distances of the FI signals across different methods.

Furthermore, as different scalings are used in the various definitions, we only report comparison results for standardized FI signals. That is, every FI time series is scaled to have zero mean and unit standard deviation.

Resampled, standardized FI signals are then compared using three metrics: the Pearson correlation coefficient ρ, the coefficient of determination *R*^2^, and the mean absolute distance MAD. For the evaluation of the metrics, the functions provided by Numpy corrcoef() (29) and Scikit-learn metrics.r2_score(), metrics.mean_absolute_error() (30) are employed. Notice that we use mean absolute *distance* rather than *error* as the latter implies the fundamental correctness of one of the signals, but this cannot be determined in the context of this work.

We compare each of presented FI implementation, to all others by a pair-wise comparison, that is quantified by the aforementioned metrics. For example, if we have five definitions of the FI_*i*_, *i* = 1, …, 5, we compute ten sets of score metrics, i.e. one for each pair of FI definitions {(FI_*i*_, FI_*j*_)|*i* = 1, …, 5∧*j* > *i*}.

As we are ultimately interested in comparing FI definitions in relation to all other definitions, we further use a leave-one-out meta-comparison. To do this, we group all scores belonging to the test definition in one set A and all scores not belonging to the test definition in another set B. We then evaluate the similarity between A and B to gauge how comparable the tested definition is in relation to all other considered definitions. We use a modified intersection over union (IOU) to assess similarity between sets. Let the range $r_{X}$ of a set X={x|x∈ℝ} be further defined as


$$\begin{array}{l} r_{X}=\left[\underset{x\in X}{\min}x,\underset{x\in X}{\max}x\right]. \end{array}$$

Let $A=\left\{a_{1},a_{2},\ldots ,a_{n}\right\}$ and $B=\left\{b_{1},b_{2},\ldots ,b_{m}\right\}$ be two sets of metrics to be compared, with *m, n* ≥ 2. We then define the union ∪(A,B) of A and B as


$$\begin{array}{l} \cup \left(A,B\right)=\underset{X\in \left\{A,B\right\}}{\max}\left\{\max r_{X}\right\}-\underset{X\in \left\{A,B\right\}}{\min}\left\{\min r_{X}\right\}, \end{array}$$

and the intersection ∩(A,B) of A and B as


$$\begin{array}{l} \cap \left(A,B\right)=\max\left\{\underset{X\in \left\{A,B\right\}}{\min}\left\{\max r_{X}\right\}-\underset{X\in \left\{A,B\right\}}{\max}\left\{\min r_{X}\right\},0\right\}. \end{array}$$

We then define the IOU of A and B as


$$\begin{array}{l} \mathrm{IOU}\left(A,B\right)=\frac{\cap \left(A,B\right)}{\cup \left(A,B\right)}. \end{array}$$

**Figure 2:**
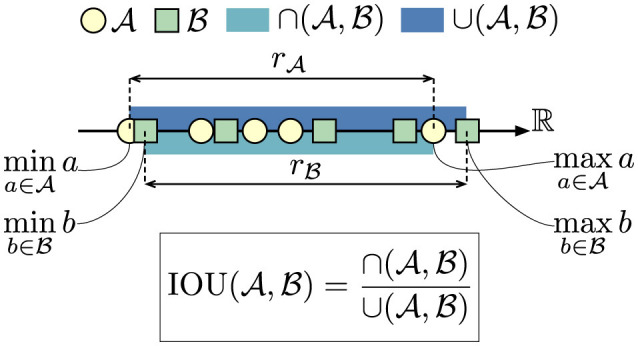
IOU variant depiction. As the elements of A and B can assume virtually any value in ℝ, the classical definition of IOU where one checks for exact matches is deemed not fitting in this context due to the fact that the elements of the sets A and B are real numbers and not integers. Hence, we work with the ranges $r_{A}$ and $r_{B}$ spanned by A and B respectively. This allows us to compare sets with differing number of elements and whose elements may assume any value in principle.

An IOU of 0 indicates no overlap between the sets, while an IOU of 1 indicates perfect overlap of the ranges of the sets. A graphical visualization of this variant of the IOU is depicted in Figure 2.

## 3 Results

Whenever real-world data is used, we show the differences between the definitions using data from the Daphnet set (15). In particular, when a single recording is used for demo purposes, the first recording of subject two (S02R01.txt) is used, as this is well-suited dataset.^3^ When not specified further, the shank's *y*-acceleration is used as a proxy, which is the “vertical” acceleration.

### 3.1 Hyperparameters tuning

The proposed multitaper FI estimation method, presented in Algorithm 1 features a number of hyperparameters that require tuning, namely the time horizon (windowing time) *T*, the number of tapers *L*, the Slepian standardized half-bandwidth *B*,^4^ the locomotion-freezing threshold frequency *f*_*t*_, and the MAF kernel size *M*.

The parameter default values used for all analyses except the hyperparameter sweep are *T* = 5.0*s*, *L* = 4, *B* = 2.5, *f*_*t*_ = 3*Hz*, as they are considered a good compromise between smoothness and responsiveness of the FI. To investigate if and to what extent each hyperparameter *T*, *L*, *B*, *f*_*t*_ affects the resulting FI, we compute the FI for a range of parameter values, while keeping the other parameters constant. The sweeping ranges for the parameters are *T* ∈ [2, 10]*s*, *L* ∈ [1, 8], *B* ∈ [0.5, 10], and *f*_*t*_ ∈ [2, 4]*Hz*.

It shall be noted that for the parametric sweep, we do not compare the standardized FI, as we are interested in showing the absolute changes in FI estimation. Standardization of the FI removes some of the differences under investigation.

**Figure 3:**
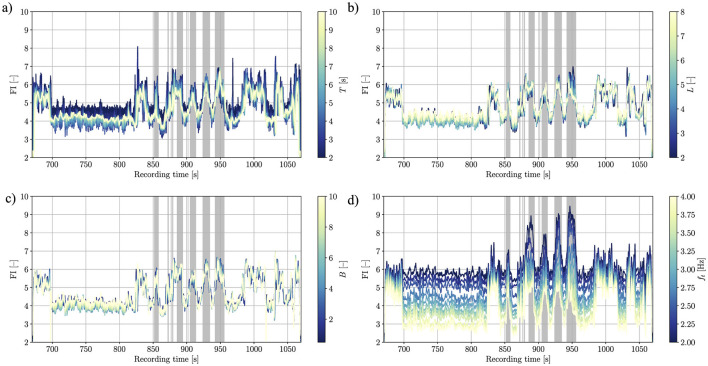
Effects of parameter sweep on FI estimation. The effects of the time horizon *T*, the number of tapers *L*, the bandwidth *B*, and the locomotion-freeze threshold frequency *f*_*t*_ are shown in **(a–d)** respectively. The gray areas indicate the FOG regions as labeled by the experts. While *T*, *L*, and *B* appear to have a modest impact on the FI, with just small changes in the smoothness/responsiveness relation (hinting toward robustness of the proposed method), *f*_*t*_ appears to have a significant effect. For *f*_*t*_ → 2.0*Hz* [darker curves in **(d)**] it can be observed that the FI appears to feature more distinguishable peaks in the actual FOG region, while staying flatter outside it.

We find that the locomotion-freezing frequency threshold *f*_*t*_ has the biggest effect on the FI. This hints toward an intrinsic robustness of the multitaper method with respect to its other hyperparameters {*T, L, B*}, which is a desired feature of the algorithm, and calls for a deeper investigation on the importance of the locomotion/freezing bands definition. It shall be noted that the results shown in Figure 3 are potentially valid only for the specific recording or even time window, as intuitively the locomotion band could be linked to one's walking cadence.

### 3.2 Comparison to existing definitions

#### 3.2.1 Performance evaluation

As shown in Table 2, the multitaper FI is the top performer among all definitions yielding the smallest variability 0.41 ± 0.05 and smallest RMSE 0.42 ± 0.05 when evaluated on white noise signals. The multitaper FI also returns the most consistent values among all definitions across all considered sampling frequencies, further strengthening the case for its use in clinical applications.

**Table 2:** Performance evaluation results across different FI definitions on Gaussian white noise.

**Sampling frequency [Hz]**	**Estimation method**	**STD**	**RMSE**
64	Moore	0.62 ± 0.06	0.64 ± 0.06
	Bachlin	1.00 ± 0.10	1.12 ± 0.13
	Cockx	1.32 ± 0.08	1.36 ± 0.10
	Zach	1.19 ± 0.07	1.21 ± 0.08
	**Multitaper (ours)**	**0.41 ± 0.03**	**0.42 ± 0.03**
100	Moore	0.64 ± 0.11	0.65 ± 0.11
	Bachlin	0.95 ± 0.19	1.03 ± 0.22
	Cockx	1.21 ± 0.10	1.22 ± 0.10
	Zach	1.14 ± 0.05	1.15 ± 0.05
	**Multitaper (ours)**	**0.41 ± 0.04**	**0.42 ± 0.04**
256	Moore	0.62 ± 0.13	0.64 ± 0.13
	Bachlin	0.96 ± 0.23	1.03 ± 0.28
	Cockx	1.24 ± 0.16	1.26 ± 0.17
	Zach	1.15 ± 0.12	1.17 ± 0.13
	**Multitaper (ours)**	**0.41 ± 0.05**	**0.42 ± 0.05**

The proposed multitaper FI shows the best performance across all tested sampling frequencies for both the variability (STD) and the RMSE. The multitaper FI also shows the most consistent results across all sampling frequencies. The shown values are the mean ± the standard deviation of the metric over the *M* = 10 simulations.

#### 3.2.2 Similarity analysis

**Figure 4:**
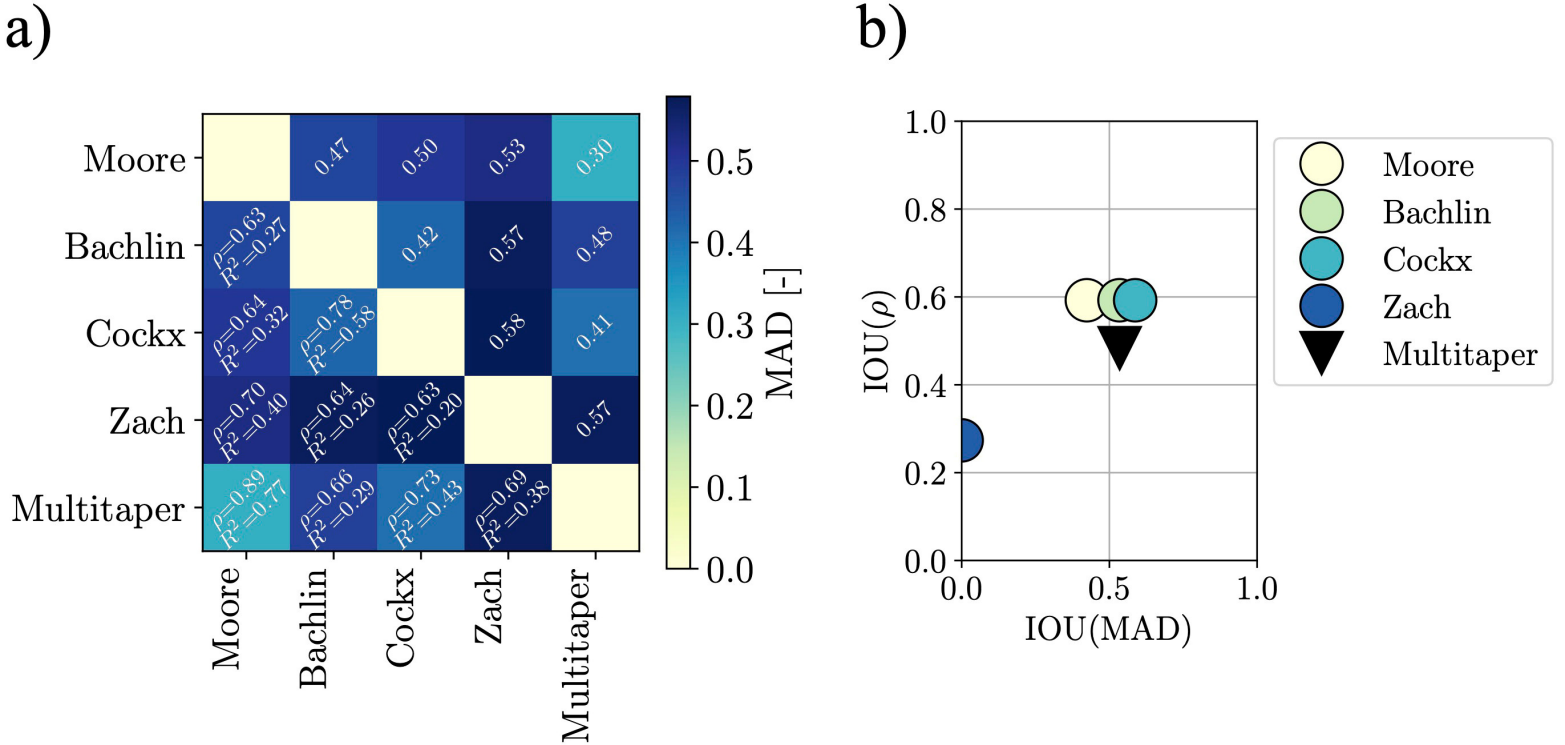
Similarity metrics for the pairwise comparison of FI estimation methods. The upper-right half of the similarity matrix reports the MAD scores, while the bottom-left half the Pearson correlation coefficient ρ, and the coefficient of determination *R*^2^ are displayed. Clearly, the Zach method differs from the others, this is visible from both the similarity matrix (darker row and column in correspondence of Zach's method) and in the IOU scatter plot (the ranges spanned by the Zach metrics have no overlap with the ranges spanned by the others). **(a)** Similarity matrix for the FI when the various implementations are used for estimation. **(b)** IOUs distribution visualization for IOUs computes with leave-one-out strategy.

Figure 4 depicts the comparison metrics and their IOUs. By comparing the literature FI definitions to the multitaper definition presented herein, we find that the introduced multitaper FI is comparable to the ones found in the literature. The IOU of the computed scores for the multitaper method are found to be approximately 0.53, 0.48, and 0.50 for MAD, ρ, and *R*^2^ respectively. By inspecting Figure 4b, it is apparent how the Zach method is the most different—this is shown by how the IOUs are clustered in the figure. Hence, we argue that the multitaper method is not an outlier among the implementations, and that its good practices and formalism make it the best candidate for a standard computation method of the FI.

### 3.3 Comparison of different proxy choices

**Figure 5:**
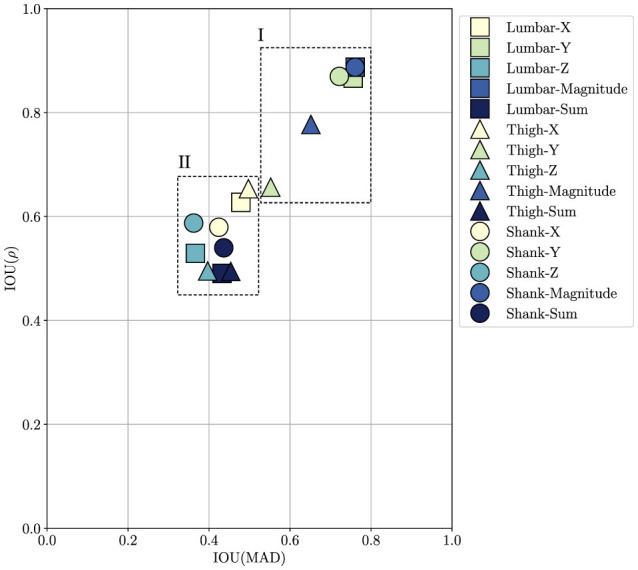
Proxy sweep IOU evaluation. IOU scattering for MAD and ρ when one proxy's similarity is compared to the similarity among all other proxies (leave-one-out strategy). The label indicates the test set, i.e., which proxy has been left out. The IOU values indicate how similar the FI for the given proxy is compared to the FI for all other proxies, relative to how similar the FI for all other proxies are compared to each other. Two groups can be separated, highlighted with the boxes I (magnitudes and *y*–direction signals) and II (others).

The test dataset (15) features lumbar, thigh, and shank accelerations in three directions *x*, *y*, *z* which are denoted as lumbar-X, lumbar-Y, lumbar-Z, thigh-X, thigh-Y, thigh-Z, shank-X, shank-Y, and shank-Z here. Two additional proxies for each sensor location are considered, namely the acceleration magnitude $a=\sqrt{a_{x}^{2}+a_{y}^{2}+a_{z}^{2}}$, and the acceleration sum *s* = *a*_*x*_ + *a*_*y*_ + *a*_*z*_. The effect of proxy choice on the FI for the test dataset is shown in Figure 5 in terms of leave-one-out IOUs. The full similarity matrix can be found in the Supplementary material. The IOU distribution shows how the proxy choice appears to have a minor effect on the FI estimation. We justify this statement by observing that the IOUs indicate how the FI computed with one proxy compares to all others in relation to how all others relate to each other, and the fact that there is no obvious pattern in the distributions of the IOUs. For all considered proxy choices, the maximum computed MAD is found to be 0.91 (lumbar-magnitude compared to lumbar-X), and the minima is 0.09 (lumbar-Y compared to lumbar-magnitude). In terms of IOU clustering, one can identify two groups of proxies: the *y*–direction signals, which are the ones biased the most by gravity, and the magnitude signals seem to form a group in terms of IOU (see Figure 4b). This is most likely due to the fact that the magnitude proxy is dominated by the *y*–direction signals, which in turn means that whenever one of these is left out for testing, the other contributes to span a similar range in the comparison set. It is somewhat surprising that the dedrifting preprocessing step does not compensate for this completely. Then again, by looking at group II in Figure 5, it is apparent that choosing a different proxy has a limited effect. Hence, the proxy choice and the method choice have similar effects on the evaluation of the FI. This justifies both the change from the original ground reaction forces (13) analysis to inertial signals, and the potential for other signals to be used to the same end, such as gyroscope measurements.

## 4 Discussion

Existing definitions of the freeze index (FI) exhibit considerable heterogeneity and frequently lack adherence to rigorous signal processing standards, limiting FI's robustness and reproducibility as a digital biomarker for freezing of gait. This inconsistency has restricted its deployment as part of remote monitoring applications for patients. Further, it is unsuitable for certified medical devices due to the absence of an established, standardized definition. To address these challenges, we introduced a robust formalism for FI computation based on multitaper power spectral density estimation, establishing a new benchmark for consistency and applicability across clinical and technological domains. To be able to expand on the FI and use it in a clinical framework, consensus on its definition is a prerequisite and establishing this is our primary contribution. Its clinical interpretation in real-world settings in relation to FOG events has to be evaluated in a clinical study. The study should focus on identifying the relationship of the FI and FOG, within the limits of the newly introduced definition of the FI. The study should also collect additional data to aid in the discrimination of confounding events such as voluntary stops and turns.

The introduced algorithm outperforms the existing definitions in terms of both error and consistency when evaluated against closed-form solutions for flat-spectrum signals as predicted by the underlying mathematical theory, and yields comparable results when evaluated on real-world data. The latter was demonstrated through similarity-analysis based on the IOU when comparing how our multitaper method compares to the existing FI estimation methods in relation to how the existing methods compare to each other when evaluated on sample data. For real-world data, the Zach method stands out as the most different from the others (smallest IOU values), and this is to be explained by its small time-horizon in combination with the differing sampling frequency between the original application (18) and the test dataset (15), as from an implementation point of view, Moore and Zach are otherwise identical. The Zach method was originally applied to signals sampled at 256 Hz, hence having 512 samples at disposal for the spectral evaluation, but the data from the Daphnet is sampled at 64 Hz, leading thus to only 128 samples being available for the FFT evaluation – this is a clear reduction of frequency resolution. Combining this fact with the absence of tapering and zero-padding as preprocessing steps yields to the Zach method differing the most from the others. This situation further underlines the need for a clear definition of how the FI is to be estimated, in addition to requiring the FI to be robust against sampling frequency changes.

While the differences between the methods have been found to be comparable across all implementations, one aspect which has not been investigated in depth yet is the definition of the frequency bands. In particular, the locomotion band has been defined as both 0–3 Hz (17) and 0.5–3 Hz (4), while the freezing band has been reported to be all of 3–6 Hz (13), 3–8 Hz (4), and 3.5–8 Hz (16). As hinted by our parametric sweep over the locomotor-freezing threshold, it may be that the introduced multitaper FI estimation algorithm shows improved performance for frequency bands specific to the underlying data, e.g. basing the threshold on the subject's cadence. A clinical study addressing this issue should be carried out in order to answer this question definitively.

When applying the methods found in literature, special care needs to be taken when deviating from the original implementation, as best signal processing practices are not fully applied. In particular, the lack of appropriate preprocessing steps (detrending, tapering, and zero-padding) means that artifacts or performance drops can appear when using the algorithm at different sampling frequencies—this is not acceptable for a standardized metric. The multitaper method with its complete preprocessing suite, is robust in this regard as demonstrated by our analysis on Gaussian signals.

The results from the real-world data need to be considered within their limitations. In particular, it shall be noted that the used dataset was limited to few subjects (*N* = 10), in a semi-controlled environment, which could have affected the participants' performance (15). The generalization of the results for the real-world application may be limited by these factors and more research should be carried out answer this question exhaustively.

When analyzing real-world data, which naturally come with high variability and unpredictability, a robust FI estimation method is imperative. Existing implementations have been shown to fall short in this sense, while the multitaper approach complies with the requirement, although this shall be formally verified in subsequent clinical trials.

Although not the primary focus of work, our investigation suggests that thresholding the FI may not be the optimal to discriminate between FOG and non-FOG episodes. FOG is a multifaceted phenomenon, and the FI barely looks at a thin slice of this complex picture—it seems overly optimistic to assign all responsibility of reliably detecting FOG in any circumstance onto the FI using simple thresholds. Fusing the unified FI implementation with other sources of information, in addition to exploring alternative classification methods, may help overcome the shortcomings uncovered in recent findings (14). This would require a proper clinical study to validate. Nonetheless, the simplicity of the FI is also one of its strengths, as it is interpretable, and its limitations are known. These aspects make it a good tool to have at hand for the monitoring of PD.

## 5 Conclusions

We have formalized the definition of the freeze index (FI) by introducing an estimation algorithm based on multitaper spectral estimation. The proposed algorithm performs on par with the existing definitions on the limited test dataset, while providing a more rigorous and reproducible foundation, made available as open source Python code, and outperforming all prior definitions when computing the performance against theoretical values.

Our study suggests that the multitaper FI method is robust across various hyperparameters such as proxy choice, and time horizon. However, to maximize reliability, a consistent/standardized use of a specific definition of the FI is to be advised. Automated FOG detection using the FI could empower clinicians to monitor and adjust treatment strategies, ultimately enhancing patient quality of life by reducing FOG symptoms. Our multitaper FI method, with its theoretical rigor, provides a valuable tool for future FOG research and clinical application.

Further investigation into optimized frequency bands for FOG detection and exploring the use of alternative sensors (e.g. gyroscope) remains a promising direction, potentially enabling more individualized and precise FI applications. Also, the general performance of the FI in real-world conditions, such as in home environments, shall be investigated in order to assess its predictive power for FOG, in particular when it is augmented with additional data such as information on turns.

## Data Availability

Publicly available datasets were analyzed in this study. This data can be found here: Daphnet Dataset, doi: 10.24432/C56K78. https://archive.ics.uci.edu/dataset/245/daphnet+freezing+of+gait.

## Footnotes

^1^As reported by the World Health Organization in 2023. Accessible online at https://www.who.int/news-room/fact-sheets/detail/parkinson-disease, accessed on 21.08.2024.

^2^In Bachlin's method no tapering is applied, hence leaving open the possibility for edge effects to arise during the FFT evaluation. In Cockx' method, the Hann window is applied to a non-zero-mean signal, which can cause undesired frequency leakage, in particular at the lower end of the spectrum.

^3^As *well-suited*, we mean that the recording includes FOG phases that are neither too many nor too frequent. Such recordings are S02R01.txt, S03R02.txt, S06R01.txt.

^4^This is the same parameter NW in the SciPy function scipy.signal.windows.dpss(). See https://docs.scipy.org/doc/scipy/reference/generated/scipy.signal.windows.dpss.html for more information – website accessed on 2024.09.11. We refer to *B* as the bandwidth in the remainder of the paper.
