# Supplementary material for: Toward a unified gait freeze index: a standardized benchmark for clinical and regulatory evaluations
Source: Front Neurol. 2025 May 8;16:1528963. doi: 10.3389/fneur.2025.1528963 (PMC12094937; doi:10.3389/fneur.2025.1528963)
Supplement: Supplementary file 1 [file Data_Sheet_1.pdf]

Supplementary Material for Towards a Unified Gait Freeze Index: A Standardized Benchmark for Clinical and Regulatory Evaluations

1 SUPPLEMENTARY FIGURES

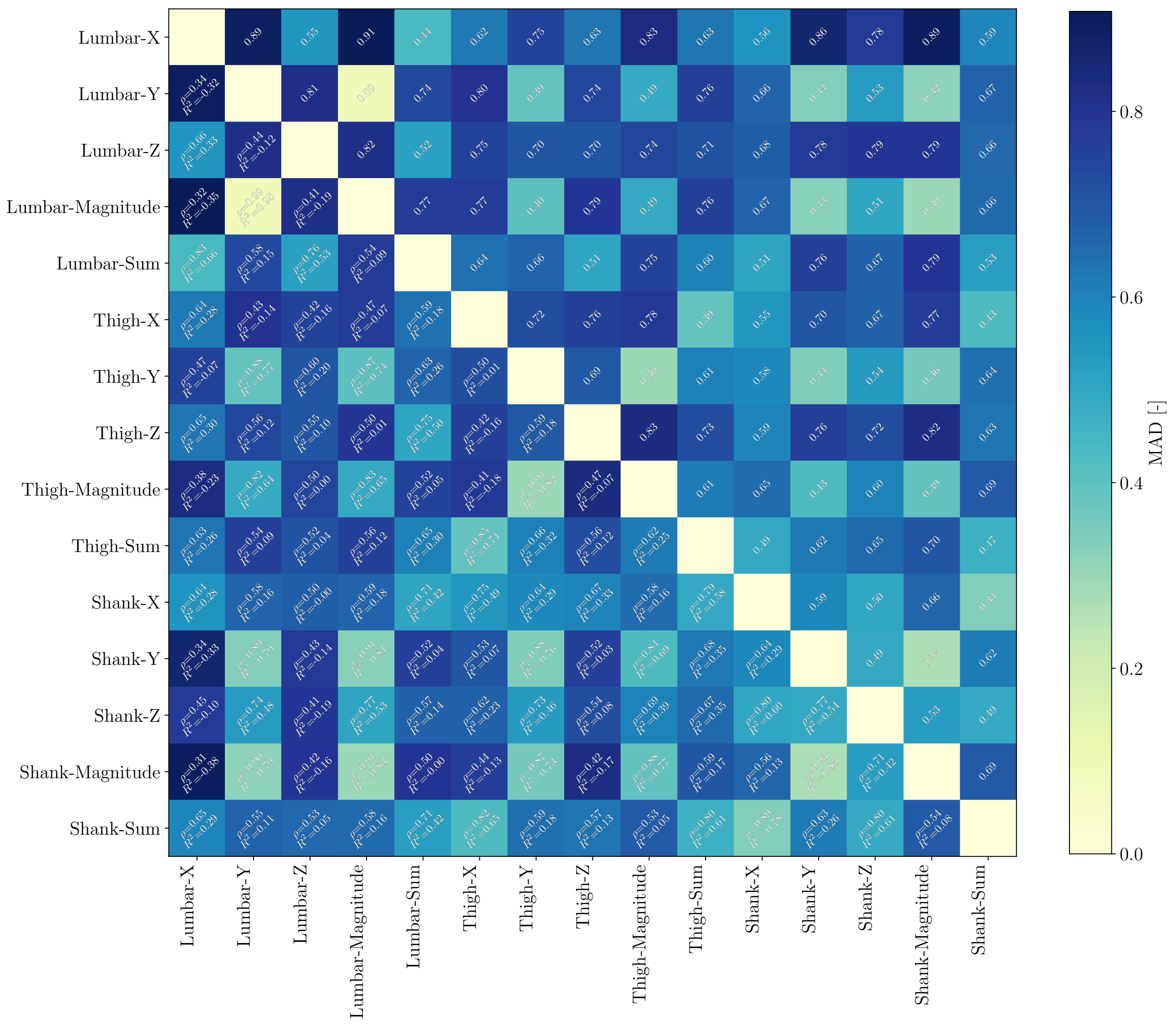

Figure S1. Proxy choice similarity analysis results. The upper-half of the similarity matrix reports the mean absolute distance (MAD) scores, while the bottom half the Pearson correlation coefficient ρ, and the coefficient of determination R^2 are displayed. The axes labels indicate the used proxy as `location-axis`.
